# The evolution of reproductive isolation in a simultaneous hermaphrodite, the freshwater snail *Physa*

**DOI:** 10.1186/1471-2148-11-144

**Published:** 2011-05-27

**Authors:** Robert T Dillon, Amy R Wethington, Charles Lydeard

**Affiliations:** 1 Department of Biology, College of Charleston, Charleston, SC 29424, USA; 2 Department of Biology, Chowan University, 1 University Place, Murfreesboro, NC 27855, USA; 3 Department of Biology, American University, 4400 Massachusetts Ave. NW, Washington, DC 20016, USA; 4 Current Address: The National Science Foundation, Division of Environmental Biology, 4201 Wilson Boulevard Arlington, VA 22230, USA

## Abstract

**Background:**

The cosmopolitan freshwater snail *Physa acuta *has recently found widespread use as a model organism for the study of mating systems and reproductive allocation. Mitochondrial DNA phylogenies suggest that *Physa carolinae*, recently described from the American southeast, is a sister species of *P. acuta*. The divergence of the *acuta/carolinae *ancestor from the more widespread *P. pomilia *appears to be somewhat older, and the split between a hypothetical *acuta/carolinae/pomilia *ancestor and *P. gyrina *appears older still.

**Results:**

Here we report the results of no-choice mating experiments yielding no evidence of hybridization between *gyrina *and any of four other populations (*pomilia, carolinae*, Philadelphia *acuta*, or Charleston *acuta*), nor between *pomilia *and *carolinae*. Crosses between *pomilia *and both *acuta *populations yielded sterile F1 progeny with reduced viability, while crosses between *carolinae *and both *acuta *populations yielded sterile F1 hybrids of normal viability. A set of mate-choice tests also revealed significant sexual isolation between *gyrina *and all four of our other *Physa *populations, between *pomilia *and *carolinae*, and between *pomilia *and Charleston *acuta*, but not between *pomilia *and the *acuta *population from Philadelphia, nor between *carolinae *and either *acuta *population. These observations are consistent with the origin of hybrid sterility prior to hybrid inviability, and a hypothesis that speciation between *pomilia *and *acuta *may have been reinforced by selection for prezygotic reproductive isolation in sympatry.

**Conclusions:**

We propose a two-factor model for the evolution of postzygotic reproductive incompatibility in this set of five *Physa *populations consistent with the Dobzhansky-Muller model of speciation, and a second two-factor model for the evolution of sexual incompatibility. Under these models, species trees may be said to correspond with gene trees in American populations of the freshwater snail, *Physa*.

## Background

Although the evolution of reproductive isolation has been a focus of intense interest since the birth of the Modern Synthesis, its quantification is often not a trivial exercise. Among animal taxa, most research effort has focused on *Drosophila *[1,2], birds [3,4], amphibians [5,6], and fish [7,8]. In gastropod mollusks, the mechanisms of reproductive isolation have been elucidated only in the marine prosobranch snail *Littorina *[9,10], opisthobranch sea slugs [11], certain terrestrial (stylommatophoran) pulmonates [12,13], and in the freshwater (basommatophoran) pulmonates that will be the focus of the present work.

The barriers to reproduction that may evolve between a pair of populations have customarily been divided into prezygotic and postzygotic components, both of which are typically inherited in a complex and polygenic fashion. Coyne [14] reported that the shortened copulations observed between *Drosophila simulans *and *D. mauritiana*, for example, are controlled by genes on all three major chromosomes, the two autosomes more important than the X chromosome. With regard to postzygotic reproductive isolation, experiments with F2 backcrosses among *Drosophila *species have consistently demonstrated that hybrid sterility is attributable to genes on every arm of every chromosome [15]. Presgraves [16] estimated approximately 191 hybrid-lethal incompatibilities between *D. melanogaster *and *D. simulans*. Thirteen loci in seven linkage groups were implicated in the origin of postzygotic isolation speciation among lake whitefish by Rogers and Bernatchez [17].

A positive correlation has often been documented between degree of reproductive isolation and overall genetic divergence [18-20]. This relationship was first demonstrated in the *D. willistoni *species complex of South America by Ayala et al. [21] using variation at allozyme-encoding loci, and has since been confirmed in *Drosophila *generally [1,2], Lepidoptera [22], fish [23], amphibians [6,24], and birds [3]. Most of these studies have focused on postzygotic reproductive isolation, which tends to evolve more slowly than prezygotic isolation [25]. Although a lag is generally noted between the accumulation of genetic differences and the onset of reproductive barriers in these studies [26], the temporal relationship between genetic divergence and reproductive isolation seems sufficiently predictable to prompt theoretical exploration of a "speciation clock" [18,27].

Since the advent of modern molecular methods for phylogenetic reconstruction, increased attention has turned toward the detailed correspondence between gene trees and the reproductive relationships among the populations from which marker genes have been sampled. As might be expected from the complex and polygenic nature of reproductive barriers, however, their correlation with any single gene or small number of genes arbitrarily selected to reconstruct a population phylogeny under the neutral model may be poor [28]. In both the *D. melanogaster *and *D. willistoni *species groups, for example, genes sampled from narrowly restricted but reproductively isolated species nest within the branches of gene trees constructed from their more widely-dispersed progenitors, rendering the ancestor species paraphyletic [25,29-31]. In the group of *D. pseudoobscura*, none of 16 genes sampled from 10-20 individuals unambiguously returned the known reproductive relationships among three populations, relative to an outgroup [32]. Such discrepancies have led some systematists to endorse "phylogenetic" species concepts based not on reproductive isolation, but rather on the evolutionary history of marker genes [33].

The purpose of the work we present here is to examine the origin of reproductive isolation in a simultaneous hermaphrodite from a phylogenetic perspective. We focus on freshwater pulmonate snails of the genus *Physa*, which by virtue of their reproductive plasticity, ease of culture, and availability of genetic markers have become important models for the study of mating systems generally [34-36]. The best-known species is *Physa acuta*, apparently a North American native introduced to Europe in the early nineteenth century and subsequently spread to six continents [37]. In cultured populations, male fertility develops around 6 weeks post-hatch, and female fertility is added around week 7, with the onset of self-fertilization around week 9, if no partner is supplied [38-41]. However, the reductions in parental fecundity and F1 viability engendered by self-fertilization seem to select strongly for outcrossing [39,42-46].

North America is also home to approximately ten lesser-known species in the family Physidae, differing from *P. acuta *by reproductive anatomy, habitat, and minor aspects of shell morphology [47]. Swamps and ditches in the southeastern United States are inhabited by *Physa carolinae*, bearing a darker and more slender shell than *P. acuta*, and river margins are inhabited by *Physa pomilia*, reaching adulthood at a smaller size [48]. Both *P. carolinae *and *P. pomilia *have two-part penial sheaths, with a larger muscular portion and a smaller glandular portion, while the penial sheath of *P. acuta *is not subdivided. Further north, more stable freshwater environments are inhabited by *Physa gyrina*, distinguished by a two-part penial sheath with approximately equal glandular and muscular portions [49,50].

In recent years substantial research effort has been directed toward quantifying the reproductive isolation among North American physid populations. The experimental tools available are directly analogous to the "mate-choice" and "no-choice" tests that have become established by common practice with dioecious animals displaying obligate sexual reproduction [33], slightly modified in their interpretation for hermaphrodites.

*Physa *mate-choice tests have been modelled closely on the experimental design used with *Drosophila *in population cages for many years [1,2,51]. Such methods have been applied to examine specific relationships in the freshwater pulmonate snail *Biomphalaria *[52,53] and in land snails [54,55], as well as in the dioecious prosobranch snail *Littorina *[56,57]. Although *Physa *are simultaneously hermaphroditic, copulation is unidirectional, with prospective partners typically vying to assume the male role. The complex behaviors documented in pairs of mating physids [58,59] have been attributed both to sexual conflict (the rejection of males by females [60]) and to gender conflict (between hermaphroditic animals vying to mate as male [61]). Thus while the reproductive relationships among pairs of physid populations can be quantified using standard mate-choice statistics, each datum is not a "choice" in any sense, but rather the outcome of a contest. Any consistent approach to scoring that outcome yielding a single observation per individual might be employed.

The use of no-choice tests to evaluate reproductive isolation was also pioneered with fruit flies [15] and such techniques have been used to explore specific relationships in the freshwater pulmonate snail *Bulinus *[62,63] and in the marine prosobranch *Lacuna *[64]. But the interpretation of such experiments is again somewhat ambiguous in hermaphrodites such as *Physa*, since any individual snail always has a "choice" to outcross with the single partner provided or to self-fertilize. Thus in addition to comparing the fertility and fecundity of outcrossed pairs to their corresponding incross controls, the hybridity of first-generation progeny must be confirmed using genetic markers, and any offspring of self-fertilization subtracted.

Here we report an expansion of our previous work to include both mate-choice and no-choice tests among all pairs of *P. acuta, P. carolinae, P. pomilia *and *P. gyrina*. We add a second population of *P. acuta *to test for discrepancies due to paraphyly, such as have been the concern of the proponents of phylogenetic species concepts. The results of these 10 possible pairwise comparisons are then cast into a phylogenic framework (a "species tree") using the mtDNA sequence data of Wethington and Lydeard [47] and Wethington et al. [48]. By this approach we are able to infer the relative rates of evolution for hybrid sterility, hybrid inviability, and sexual incompatibility.

## Results

### Sexual isolation

As has previously been documented [45,58,59], our approach of rearing snails to adulthood in isolation before initiating mate-choice tests seemed to yield many animals eager to copulate in the male role, while rejective in the female role. We typically observed a high frequency of mating activity over the six hours of each trial, each snail often involved in multiple copulations. Across the ten pairwise comparisons of our five populations, the minimum number of initial copulations as male observed was 38, and the median 47, of 60 possible (Table 1).

**Table 1:** Results of mate choice tests

	*acuta*-c	*acuta*-p	*carolinae*	*pomilia*	*gyrina*
*acuta*-c	-	52	49	41	43
*acuta*-p	0.19	-	44	50	41
*carolinae*	0.13	0.23	-	38	51
*pomilia*	0.46*	0.01	0.45*	-	54
*gyrina*	0.71*	0.51*	0.51*	0.37*	-

Below the diagonal are values for the IPSI of Rolán-Alvarez and Caballero [78] testing for sexual isolation in 2 × 2 tables of mating frequency among all pairs of five *Physa *populations, and above the diagonal are the grand totals of the copulations upon which the statistic is based, of 60 maximum.

*Significant at the tablewide 0.05 level by Bonferroni correction.

Values of the IPSI statistic testing for sexual isolation between all pairs of populations are given below the diagonal of Table 1. No sexual isolation was demonstrated in pairings among *Physa carolinae *and *Physa acuta *(either Charleston or Philadelphia), all copulations involving those three comparisons appearing indiscriminate. Our experiments did generally detect significant sexual isolation between populations of the *acuta/carolinae *group and both *Physa pomilia *and *P. gyrina*. The exception was the *acuta*-p × *pomilia *comparison, where 14 of the 22 initial copulations of Philadelphia *acuta *as males were with *pomilia *as females, and 10 of the 28 initial copulations of *pomilia *as males were with *acuta *as females, yielding an IPSI statistic near zero.

### Hybridization

**Figure 1:**
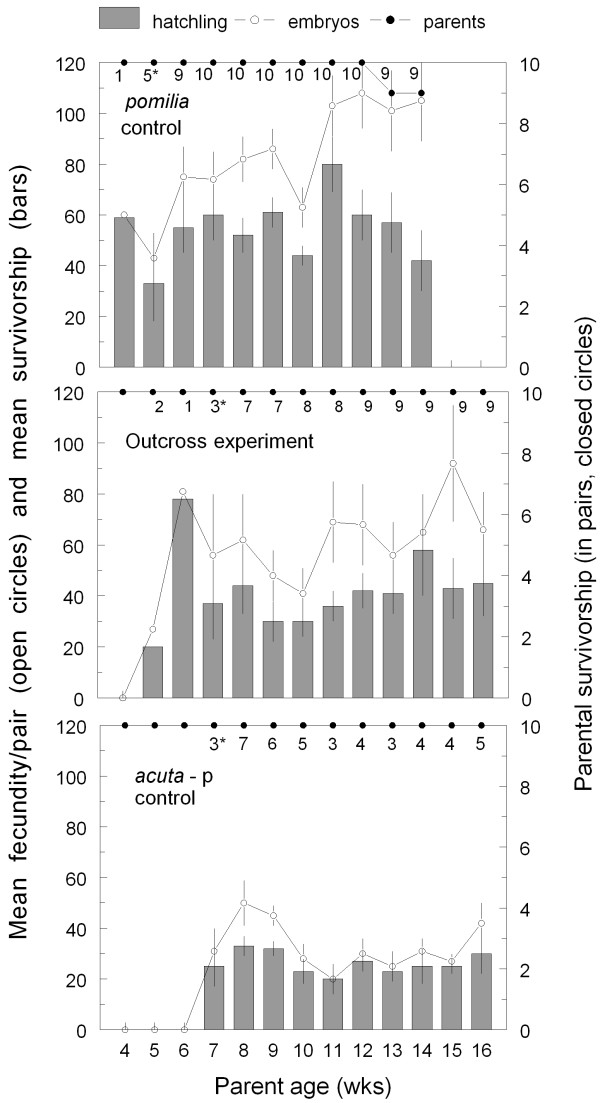
**Example results from a no-choice experiment testing for postzygotic reproductive isolation**. Along with parental survivorship (right axis, solid circles) we report the number of reproducing pairs, an asterisk denoting week 1 for comparisons of parental fecundity and F1 viability. Mean offspring per pair (left axis) shows both fecundity plotted as open circles and offspring survivorship as a bar graph. The bars are standard errors of the mean.

Example results from one of our no-choice experiments and its corresponding pair of controls are shown in Figure 1. Parental survivorship was excellent in this set of 30 pairs, as was generally observed through the duration of all experiments and controls reported here, only one pair of parents failing to survive the 16 weeks of observation. In this case the *pomilia *controls initiated reproduction at an earlier week (and at a smaller size) than either the *acuta*-p controls or the *acuta*-p × *pomilia *outcross experiment, the three-pair threshold being reached at week 5 for *pomilia*, but week 7 for *acuta*-p and *acuta*-p × *pomilia*. Then through the ten subsequent weeks, Figure 1 demonstrates that parental fecundity was greatest in the *pomilia *control, lowest in the *acuta*-p control, and intermediate in the outcross experiment, with the survivorship of the F1 hatchlings roughly comparable in all three cases. Oviposition was ultimately observed in 9 outcross pairs, 7 control *acuta*-p pairs, and all 10 control *pomilia *pairs.

Age of first parental reproduction, parental fecundity, and the yield of viable F1 hatchlings in all no choice experiments across all three years of experimentation are compared with their corresponding controls in Table 2. Note that statistics shown in the lower half of each table section should be compared to the corresponding controls leftward of the dot, and statistics in the upper half of each section to corresponding controls right of the dot. We were unable to confirm any outcross progeny among the F1 recovered from five no-choice experiments - the four involving *Physa gyrina *and the one pairing *P. carolinae *with *P. pomilia*. Allozyme electrophoretic analysis of the first generation progeny recovered from these sets of outcross pairs revealed all reproduction to have been the result of self-fertilization by the non-*gyrina *parent in the *acuta*-p × *gyrina *and *carolinae *× *gyrina *experiments, as was previously reported in the *acuta*-c × *gyrina *experiment by Dillon et al. [65]. Only the *carolinae *parent reproduced in the *carolinae *× *pomilia *experiment. Both parents self-fertilized to produce the first generation offspring recovered from the *pomilia *× *gyrina *experiment.

**Table 2:** Results of no-choice experiments

		*acuta*-c	*acuta*-p	*carolinae*	*pomilia*
1st ovi.	*acuta*-c	8 (5-10) •			
	*acuta*-p	4 (3-5)	3 (3-4) • 8 (7-9)	8 (7-9)	8* (5-12)
	*carolinae*	7 (7-10)		8 (7-8) • 8 (7-12)	
	*pomilia*	9 (7-16)		Selfing only	7 (7-11) • 6 (4-7)

Par rep.	*acuta*-c	10/10 •			
	*acuta*-p	10/10	10/10 • 7/10	10/10	9/10
	*carolinae*	9/9		8/8 • 10/10	
	*pomilia*	8/9		Selfing only	9/9 • 10/10

Par fec.	*acuta*-c	67 (17-104) •			
	*acuta*-p	42 (21-70)	55 (26-81) • 33 (19-41)	77 (28-163)	60 (9-115)
	*carolinae*	55* (18-81)		75 (19-105) • 117 (82-153)	
	*pomilia*	25 (3-96)		Selfing only	57 (19-68) • 81 (50-121)

F1 viab.	*acuta*-c	.62 (.45-.86) •			
	*acuta*-p	.83 (.68-.93)	.87 (.66-.95) • .76 (.51-.95)	.54 (.31-.65)	.71^M ^(.58-.83)
	*carolinae*	.73 (.49-.99)		.81 (.66-.91) • .36 (.24-.55)	
	*pomilia*	.67^M ^(0.0-.91)		Selfing only	.56 (.33-.86) • .67 (.56-.83)

Modal week of first oviposition, maximum parental reproduction (as a fraction of survivorship), median weekly mean parental fecundity, and median weekly mean F1 viability (ranges) in the subsets of experiments ultimately yielding hybrid F1 progeny.

Results shown to the left and below the dotted diagonals (experiments and their corresponding controls) are from 2001 or 2002. Results from 2003 are shown to the right and above the diagonal.

*Delays or reductions significant at the tablewide 0.05 level by Bonferroni correction.

^M ^Mixtures of selfed and outcross progeny.

Two sets of no-choice mating experiments yielded F1 progeny that proved to result from mixtures of outcrossing and self-fertilization - the crosses of *Physa pomilia *with *Physa acuta *from both Philadelphia and Charleston (marked "M" in Table 2). Parents in the *acuta*-p × *pomilia *experiment exhibited a significant delay in reproduction (Figure 1), although no such phenomenon was detected in the *acuta*-c × *pomilia *experiment (Table 2 upper). Nor was any significant reduction apparent in mean parental fecundity (Table 2 middle) or the combined survivorship of the (heterogeneous) F1 progeny (Table 2 lower) from either outcross experiment. Nevertheless, a fixed difference at the LAP locus allowed Dillon et al. [66] to unambiguously classify 36 F1 progeny analyzed from the *acuta*-c × *pomilia *experiment as 25% self-*acuta*, 25% self-*pomilia *and 50% outcrossed. Polymorphisms at the LAP, Isdh, Est6 and Pgm loci permitted us to classify 68 F1 progeny recovered from the *acuta*-p × *pomilia *experiment (newly reported here) as 25% self-*acuta*, 56% self-*pomilia*, and 19% outcrossed.

Our second generation observations further suggested that the subsets of hybrid progeny recovered from both of the *acuta *× *pomilia *outcross experiments may have been sterile. Only four of the nine F1 × F1 pairs from the *acuta*-c × *pomilia *experiment yielded any viable second generation progeny after 21 weeks of observation, all four of which were subsequently revealed by allozyme analysis to have involved selfed F1 animals [66]. Six of the nine F1 × F1 pairs tested from the *acuta*-p × *pomilia *experiment ultimately yielded viable progeny in the second generation, the slightly higher proportion attributable to the lower proportion of hybrids among the F1.

Allozyme analysis confirmed that the three remaining no-choice experiments (involving *acuta*-c, *acuta*-p, and *carolinae*) yielded entirely hybrid F1 progeny, with viabilities intermediate between their two corresponding incross controls (Table 2, lower). All nine of the F1 × F1 crosses from the *acuta*-c × *acuta*-p experiment yielded viable F2 progeny, with no apparent delay in reproduction [37]. Although oviposition was noted in six of the nine F1 × F1 pairs from the *acuta*-p × *carolinae *experiment, however, only one pair yielded viable second generation progeny over 21 weeks of observation. And none of the nine F1 × F1 pairs tested from the *acuta*-c × *carolinae *experiment yielded any viable F2 offspring over 12 weeks of observation, although again, oviposition was observed in 8 pairs [67]. It would appear that *acuta x carolinae *hybrids are almost entirely sterile.

### Molecular phylogeny

**Figure 2:**
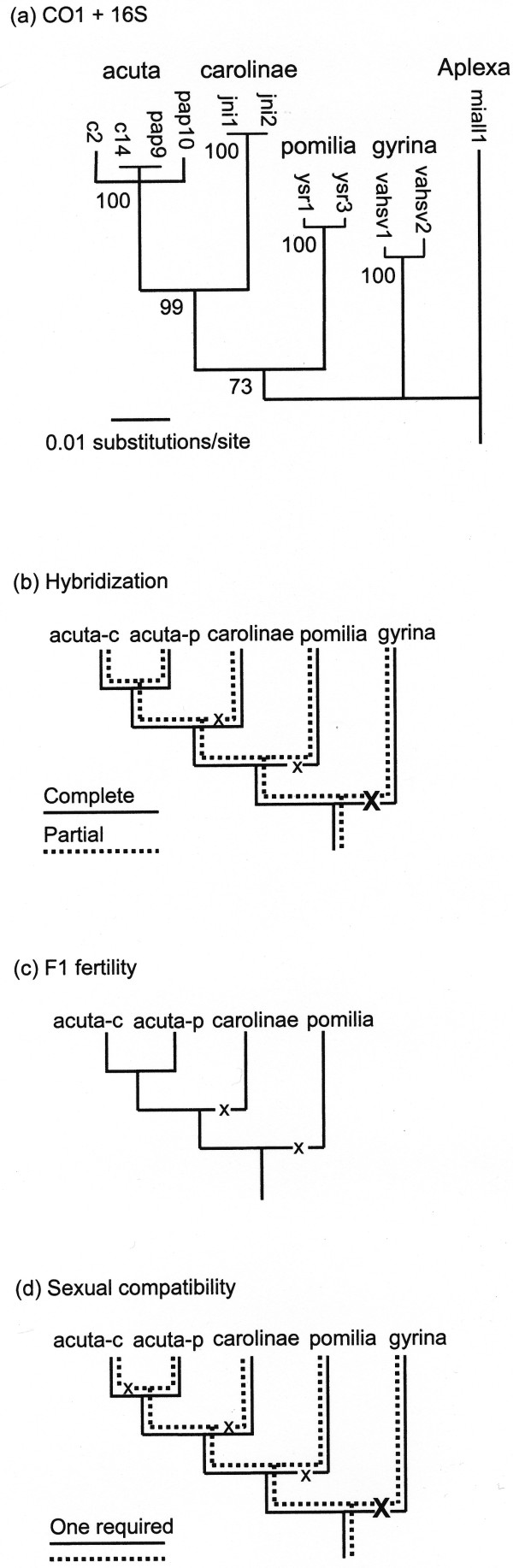
**Gene trees and species trees**. (a) Gene tree based on mitochondrial COI and 16S sequences from representative individuals of 5 *Physa *populations, with *Aplexa *as outgroup. Jackknife values are given at the nodes. For sequence codes and genbank numbers see Wethington and Lydeard [47] and Wethington et al. [48]. (b) Species tree modelling hybrid viability. The solid line is a hypothetical locus "J," a match at which allows unimpeded hybridization, and the dashed line a second locus "K," a match at which allows partial hybridization. (c) Species tree modelling the evolution of F1 fertility as a single locus. (d) Species tree modelling mating compatibility. Two loci are hypothesized, a solid "J" and a dashed "K," a match at only one of which is sufficient for unrestricted mating.

The strict consensus tree from our phylogenetic analysis of mtDNA sequence divergence is shown in Figure 2a, branch lengths scaled by neighbor-joining. Of the 979 characters on which this tree was based, 224 were variable and parsimony-informative, and 68 were variable but uninformative. This simplified tree agrees well with the more comprehensive phylogenies published by Wethington and Lydeard [47] and Wethington et al. [48], *Physa acuta *and *P. carolinae *being depicted as sister taxa, with *P. pomilia *their most immediate joint ancestor, and *P. gyrina *more distant, given *Aplexa *as an outgroup.

## Discussion

Although it is not conceptually difficult to model the process of biological speciation as a phylogenetic tree, graphically the exercise may pose substantial challenges. This is because the data are relational. Reproductive isolation is not a character that can be attributed to a population, but rather can only be measured among pairs of populations.

At both the finest level and at the most coarse, however, the observations we report here on reproductive isolation among our five *Physa *populations match the consensus gene tree returned by our phylogenetic analysis unambiguously. Mitochondrial genes sequenced from individuals sampled from our two *P. acuta *populations were the most similar molecularly, and those two populations displayed no reproductive isolation detectable by our methods. And our *P. gyrina *population was the most reproductively isolated of the five populations we examined, and contained the two individuals whose mtDNA sequence was the most divergent in our molecular phylogeny. No viable hybrids were produced when *gyrina *was paired with any other population; individual *P. gyrina *strongly preferred to copulate among themselves if offered a choice.

A simple model for the evolution of F1 infertility among the four populations from which any *bona fide *hybrids were recovered is offered in Figure 2c. This process can be cast directly on the gene tree (Figure 2a) by assuming a single compatibility locus, with some unique allele for incompatibility evolving on two separate occasions.

At intermediate levels of population divergence, however, the correspondence between our gene tree and the evolution of reproductive isolating mechanisms is less clear. For while the cross of *carolinae *to *acuta *(either Charleston or Philadelphia) yielded 100% hybrids in high frequencies, and the cross of *pomilia *to *acuta *(either population) yielded hybrids in reduced frequencies (mixed with selfed progeny), no viable *carolinae *× *pomilia *hybrids were recovered. No rearrangement of the phylogeny implied by our gene tree can account for such observations using a single factor. Similarly, our mate-choice data do not cast easily onto the gene tree phylogeny at intermediate levels of divergence. For although we detected significant sexual isolation between the *acuta/carolinae *group and both *pomilia *and *gyrina *in almost all combinations, populations of *P. acuta *from Philadelphia and *P. pomilia *seemed to copulate freely.

In Figure 2b we offer a two-locus model that might account for our observations on hybridization among our five populations of *Physa*. We postulate a "complete" locus J and a "partial" locus K, at which unique compatibility alleles 1, 2, 3, ... may segregate. Locus J is epistatic over locus K in our model, such that hybrids homozygous at the J locus are always completely viable, regardless of genotype at locus K, but J heterozygotes may show either partial viability (homozygous K) or complete inviability (heterozygous K). Thus from a J^1^K^1 ^ancestor, *gyrina *might be modelled as fixed for J^2^K^2^, *pomilia *might be J^3^K^1^, *carolinae *might be J^1^K^3^, and both *acuta *populations retain the ancestral J^1^K^1 ^genotype. Interaction between a pair of genes in this fashion would be consistent with the classic Dobzhansky-Muller model of speciation [33]. We emphasize that we have no data directly bearing on the genetics of reproductive isolation in *Physa*, however, and that other models might be offered to fit our observations on hybrid viability equally well.

Nor can any single-factor model account for our observations on prezygotic reproductive isolation among our five populations of *Physa*. But again, a two-locus model such as that offered in Figure 2d would account for our observations, consistent with the gene tree. Here we postulate two complementary loci J and K, with unique compatibility alleles 1, 2, 3,..., a match at one locus being sufficient for sexual compatibility. Then from a J^1^K^1 ^ancestor, under our model *gyrina *might be J^2^K^2^, *pomilia *might be J^3^K^1^, *carolinae *might be J^1^K^3^, *acuta*-c might be J^1^K^4^, and *acuta*-p might retain the ancestral genotype J^1^K^1^.

Our observations are consistent with at least one generalization that has emerged from comparative studies of speciation in many other groups of organisms - that hybrid sterility evolves faster than hybrid inviability [33]. While hybrids between *acuta *and *carolinae *were entirely viable, and hybrids between *acuta *and *pomilia *partially so, the measured levels of fertility in all these hybrid classes were effectively nil. Regarding the relative rates of evolution for hybrid inviability and sexual incompatibility, our results seem to favor a slightly earlier appearance of the latter. Although neither form of reproductive isolation appeared effective at the branch between *carolinae *and the Charleston/Philadelphia *acuta *ancestor, our observations at the branch between *pomilia *and the joint *acuta*/*carolinae *ancestor suggest that evolution at one (hypothetical) locus for sexual compatibility may have occurred only quite recently.

Taken together, our data are consistent with the hypothesis that speciation between *P. acuta *and *P. pomilia *may have been reinforced by natural selection for prezygotic isolating mechanisms [68,69]. Although *P. acuta *ranges throughout North America, *P. pomilia *seems more common and widespread in the American southeast. In the Charleston area populations of both species are not uncommonly sympatric, especially in the backwaters or margins of slow-moving rivers. We are not aware of any populations of *P. pomilia *in the vicinity of Philadelphia, however. Given the significant delay in reproduction we documented in the *acuta*-p/*pomilia *outcross experiment (Figure 1, Table 2 upper), the high frequency of selfed progeny among their F1 (Table 2 lower), and the apparent sterility suffered by their hybrids, one might expect selection for prezygotic reproductive isolating mechanisms in sympatry, such as those returned from our mate-choice experiments between *pomilia *and Charleston *acuta *(Table 1). But the evolution of prezygotic isolation would seem less likely between *pomilia *and allopatric Philadelphia *acuta*, where no such selection may have taken place.

Although tabulated as "choices," the primary data we recorded from our mate-choice tests were each the outcome of a contest [61]. The 41 copulations we observed in the *acuta*-c × *pomilia *test included just 11 heterogametic pairings, equally split by gender - 5 with Charleston *acuta *serving as the female and 6 with *pomilia *the female [66]. The 50 copulations we observed in the *acuta*-p × *pomilia *test included 24 heterogametic pairings, also evenly split by gender. Thus our data contain no overt evidence of differing selection regimes in the *P. acuta *populations of Philadelphia and Charleston. And it must be emphasized that a variety of explanations other than reinforcement have been offered to account for differing degrees of prezygotic reproduction among species in sympatry and allopatry [33].

## Conclusions

Our observations on the evolution of reproductive isolation in our five populations of *Physa *can be modelled with five genes - two loci for sexual compatibility, two loci for hybridization, and one locus for F1 fertility. The trees depicting evolution at these five loci (Figures 2d, b, and 2c) are consistent with the gene tree shown in Figure 2a.

We note, however, that some confusion has arisen in recent years over the term, "species tree. "Although the concept has an old history, "species tree" was brought into modern prominence by Maddison [70] to distinguish a phylogeny based on genes (a "gene tree") from the true evolutionary history of a set of populations, which he called a "species tree. "Although the example postulated by Maddison ("when reproductive communities are split by speciation") implied reproductive isolation, the processes he highlighted that might lead to discord ("lineage sorting or deep coalescence") would apply to any set of populations regardless of their reproductive relationships. Thus many subsequent workers (e.g. [71,72]) have used the term "species tree" as a synonym for "population tree."

But biological species are typically composed of more than one population, and the complex reproductive relationships among a set of populations will have their own evolutionary history, independent of (although probably correlated with) both the true historical relationships of the population tree and whatever molecular markers systematic biologists might find convenient to construct gene trees. We would suggest that the term "species tree" be reserved for figures conveying the evolution of reproductive isolation, such as those shown on Figures 2b, c and 2d, and that the term "population tree" be applied by workers such as Maddison. And then under the narrower definition adopted here, gene trees correspond to species trees in the freshwater snail, *Physa*.

## Methods

### Study populations

The five populations from which our samples were drawn were all apparently large and open, certainly numbering in the millions of individuals. The population of *Physa acuta *here abbreviated "*acuta*-c" originated from the pond at Charles Towne Landing State Park, Charleston, SC (32.8062°N; 79.9862°W). This is the standard population from which many of our laboratory lines have originated for previous studies of *Physa *reproductive biology [37-39,61,67,73]. The population of *P. acuta *abbreviated "*acuta*-p" was originally sampled from the Schuylkill River in Philadelphia, PA (39.9951°N; 75.1923°W). This is the type locality of *Physa heterostropha*, synonymized under *P. acuta *by Dillon et al. [37]. Our sample of *Physa carolinae *originated from the spring at Huger Landing in Berkeley Co, SC (33.1305°N; 79.8111°W). This is the type locality for the species [48]. We collected our population of *Physa pomilia *from the Combahee River at Yemassee, SC (32.7060°N; 80.8281°W). This is the type locality of *Physa hendersoni*, synonymized under *P. pomilia *by Dillon et al. [66]. Our population of *Physa gyrina *originated from Hot Springs, VA (38.0012°N; 79.8330°W). This is the type locality of *Physa aurea*, subsequently synonymized under *P. gyrina *[65,74,75].

### Culture

Our standard culture vessel was a transparent polyethylene 10 oz. drinking cup, filled with approximately 210 mL of aerated, filtered pond water, and covered with a 95 × 15 mm polystyrene Petri-dish lid. The food was O.S.I. Spirulina Aquarium Flake Food (Ocean Star International, Hayward, CA), finely ground. All experiments were conducted at room temperature, approximately 23°C, in a 12:12 diurnal cycle. We initially isolated 10 wild-collected snails from each study population in separate cups, collected egg masses with weekly feeding and water change, and reared the offspring to 2-mm shell length, well in advance of maturity. These unrelated sets of wild-conceived but laboratory-born sibships were designated carolinae1 through carolinae10, pomilia1 through pomilia10, gyrina1 through gyrina10, and so forth. From these five sets of ten sibships we drew isolates for mate-choice tests and pairs of parents for no-choice hybridization studies.

### Mate-choice tests

For mate-choice tests, large samples of juvenile snails from all populations were isolated in individual cups and reared to maturity over the course of 8 to 10 weeks, with weekly feeding and water change. Then for each of the 4+3+2+1 = 10 pairwise comparisons (*carolinae *× *pomilia, carolinae *× *gyrina, pomilia *× *gyrina*, and so forth) we performed 3 mating trials, each trial involving 10 adults from two populations. The 20 snails selected for each trial were all approximately equal in shell size, to minimize size-dependent gender effects [76,77]. Each was blotted dry and marked with a small dab of fingernail polish according to its population of origin. Then all 20 individuals were simultaneously introduced into a 2-L glass beaker (filled with 1,400 mL of filtered, aerated pond water) and placed on a glass table to facilitate observation.

We monitored mating activity for 6 hours. When a snail first successfully copulated as male (defined as the complete insertion of its penis into the gonopore of a partner), it was removed from the beaker, its shell marked with a dot of white correction fluid, and returned. Each individual snail was often involved in many matings over the 6 hours of observation, in both the male and the female role, but only its first successful copulation as a male was recorded. Note that this design yields a slight bias toward heterogametic pairings, not 1:1 but rather 9:10.

Each of the three trials for all ten pairwise comparisons involved 20 fresh, unmated snails. Fisher exact tests (with Bonferroni correction) returned no evidence of significant heterogeneity across the three trials for each comparison. Thus we pooled our results across trials to yield mate-choice observations on 30 snails from each population combined with 30 snails from each of the other four populations. The Rolán-Alvarez and Caballero [78] Index of Pair Sexual Isolation (IPSI) was calculated from each of the ten 2 × 2 frequency tables that resulted using JMating 1.0.8 [79], and tested with t-statistics, using sequential Bonferroni correction. Of the many statistics commonly employed to measure sexual isolation, only the IPSI demonstrates negligible bias from differing mating propensity at lower sample sizes [80]. Given the prediction that speciation will yield positive-assortative mating, one-tailed tests were appropriate.

### No-choice tests

No-choice experiments were set up with ten unrelated juveniles (at size 2 mm) paired between all populations, for example carolinae1 × pomilia1, carolinae2 × pomilia2, ..., carolinae10 × pomilia10. Two corresponding sets of controls were initiated using ten pairs of juveniles from unrelated sibships within each population, for example carolinae1 × carolinae2, carolinae2 × carolinae3, ..., carolinae10 × carolinae1 and pomilia1 × pomilia2, pomilia2 × pomilia3, ..., pomilia10 × pomilia1. Each of these 30 pairs of snails (designated the parental generation) received a water change and fresh food every 7 days, at which time the sides of the cup were inspected for egg masses. If egg masses were present, we counted all embryos as a measure of fecundity and transferred the parents to a fresh cup. Eggs were monitored until hatching (generally about 2 weeks) and all viable, crawling F1 juveniles were counted. Observation was terminated upon the death of either parent in a pair. The total period of observation varied for each set of ten pairs, as detailed below.

Evidence of speciation will be demonstrated ifthe central tendency of age at first reproduction in any set of 10 experimental pairs is significantly delayed behind the slower of its two sets of 10 corresponding control pairs. This (one-tailed) hypothesis was tested between each pair of populations by calculating a combined (20 pair) median and comparing counts above and below that median using a Fisher’s exact test, with Bonferroni correction.

For an independent analysis of fecundity and F1 viability, week 1 was established separately for each set of 10 pairs as the first week in which eggs were laid by 3 or more pairs of parents. Embryos and viable hatchlings were subsequently counted for 10 weeks. We then averaged the embryo production of each pair of parents across its lifetime, ignoring any leading (pre-maturity) zeros and any postmortem zeros, while tallying as “0” any failure to reproduce by viable, mature pairs. We used Kruskal-Wallis nonparametric ANOVAs to test the one-tailed hypothesis that the central tendency in the weekly mean fecundity of each set of 10 experimental pairs might be depressed below its two corresponding sets of 10 control pairs, with Bonferroni correction.

Similarly, we averaged the counts of F1 hatchlings within pairs across weeks, ignoring zeros not corresponding to embryo production, and divided each pair mean by its mean embryo production to obtain pair mean F1 viability. We used a second set of Kruskal-Wallis nonparametric ANOVAs to test the one-tailed hypothesis that the central tendency in the weekly mean F1 viability posted by each set of 10 experimental pairs might be depressed below its 2 corresponding sets of 10 control pairs, with Bonferroni correction.

To assess the fertility of putative hybrid offspring, F1 hatchlings (both experimental and control) were reared from each of 3 separate unrelated pairs to a size of 2 mm. These were crossed in time series: 1 early pair from eggs laid around week one, 1 middle pair produced around week five, and 1 late pair produced around week ten, to yield 9 F1 pairs. So (for example) if the putative hybrid progeny were reared from pairs carolinae1 × pomilia1, carolinae2 × pomilia2, and carolinae3 × pomilia3, they were crossed as CP1 × CP2 early, CP2 × CP3 early, CP3 × CP1 early, CP1 × CP2 middle, CP2 × CP3 middle, ..., CP3 × CP1 late. Nine crosses were likewise constituted for controls (*carolinae *and *pomilia *in this example), and the total of 3 × 9 = 27 crosses of F1 snails reared to adulthood for each experiment, with weekly feeding and water change. We recorded the dates at which embryos and viable F2 hatchlings were produced by each of the 27 pairs of F1 snails, for all 4 + 3 + 2 + 1 = 10 pairwise comparisons between populations.

### Allozyme analysis

A larger sample of F1 progeny from the 3 experimental pairs (for example, carolinae1 × pomilia1, carolinae2 × pomilia2, and carolinae3 × pomilia3, following as above) were reared to 4-mm to 5-mm shell length, at which time they were frozen in 100 μL of tissue buffer for analysis by allozyme electrophoresis to confirm hybridity. We have identified 12 enzyme-encoding loci at which allozyme variation is interpretable as the product of codominant alleles segregating in Mendelian fashion [81]. These are aconitase (Acon), esterases (three loci: Est1, Est3, Est6), glucose phosphate isomerase (Gpi), isocitrate dehydrogenase (two loci: Isdh1 and Isdh2), leucine aminopeptidase (Lap), mannose phosphate isomerase (Mpi), phosphoglucomutase (2 loci: Pgm1 and Pgm2), and 6-phosphogluconate dehydrogenase (6pgd). We used horizontal starch gel electrophoresis in an aminopropylmorpholine pH 6 buffer system to resolve allozyme variation at the Gpi, Isdh, and 6pgd loci; a Tris-Citrate pH 6 buffer system for Acon, Mpi, and Pgm; and a TEB8 system for 6pgd, Lap, and Est. Details regarding our electrophoretic methods, including a description of our equipment and recipes for stains and buffers, have been previously published [82,83].

### Overall analysis

The data on reproductive isolation analyzed here were gathered in three sets, fresh controls being required with each set. The first set of experiments, no-choice tests of hybridization between *P. acuta *from Philadelphia (*acuta*-p) and Charleston (*acuta*-c), were completed in 2001 and published (together with data on four other *P. acuta *populations) by Dillon et al. [37]. The fecundity and F1 viability data reported in that work are here trimmed back to a 10-week basis for comparison, medians and ranges being substituted for means and variances. The second set of experiments, both mate-choice tests and no-choice experiments involving *acuta*-c, *gyrina, carolinae*, and *pomilia*, were conducted in 2002. Our *acuta*-c × *gyrina *results were published by Dillon et al. [65], our *acuta*-c × *pomilia *results (confirming the specific status of *P. pomilia*) were published by Dillon et al. [66], and our *acuta*-c × *carolinae *and *acuta*-c × *pomilia *results (confirming the specific status of *P. carolinae*) were published by Dillon [67]. Our 2002 *gyrina *× *carolinae *and *gyrina *× *pomilia *results have not heretofore seen publication. The third set of experiments, both mate-choice and no-choice tests among populations *acuta*-p, *gyrina, carolinae*, and *pomilia*, were conducted in 2003 and have been entirely unpublished until the present.

We have also published several large scale or comprehensive molecular phylogenetic analyses of the Physidae in recent years [47,48,74]. The simplified gene tree extracted for the present work was based on a concatenation of a 633 base pair segment of the mitochondrial cytochrome oxidase subunit I gene and a 346 base pair segment of the mitochondrial 16S rRNA gene (loops removed). Sequences were available for two individuals from each of our five populations, which we analyzed by standard heuristic search with optimality criterion set to parsimony, rooting with the outgroup physid *Aplexa elongata *(PAUP* version 4.0b10 [84]). The strict consensus tree recovered was evaluated with a jackknife procedure (10,000 reps with 50% deletion each rep), and its branch lengths scaled using a neighbor-joining analysis.

The topology of the gene tree recovered was used as a starting point for models reconstructing the evolution of reproductive isolation in our sample of five *Physa *populations. We assumed reproductive compatibility at the base of the tree, and hypothesized a minimum number of independent origins for reproductive barriers on the branches.

## Competing interests

The authors declare that they have no competing interests.

## Authors' contributions

RTD designed the study, performed most of the experiments and analyses and drafted the manuscript. ARW performed some of the experiments and the molecular analysis. CL was the PI/PD for the overall project. All authors have read and approved the final manuscript version.
